# Huge pilomatrixomas of the scalp: A case report

**DOI:** 10.1016/j.ijscr.2021.106048

**Published:** 2021-05-29

**Authors:** Omar Wydadi, Walid Bijou, Mohammed Laachoubi, Youssef Oukessou, Mohammed Roubal, Mohammed Mahtar

**Affiliations:** a ENT Department, Face and Neck Surgery, Hospital August, 20, 1953, University Hospital Center IBN ROCHD, Casablanca, Morocco; b Faculty of Medicine and Pharmacy, Hassan II University of Casablanca, B.P 5696, Casablanca, Morocco

**Keywords:** Pilomatrixomas, Tumor, Skin, Case report

## Abstract

Pilomatrixoma is a rare benign skin tumor differentiating toward hair matrix cells usually encountered in the head and neck region. It is most frequently appearing in the first and second decades of life. Histopathological examination is essential to make definitive diagnosis. Herein, we present an atypical case of multiple pilomatrixomas. A 69-year-old man with multiple voluminous masses over the scalp. Among the three lesions, one was clinically suspicious for malignancy, it measured 17 cm and was ulcerated in places. Histopathology confirmed the diagnosis of pilomatrixoma. The tumors were removed surgically with free margins. Otolaryngologist should be familiar with this benign tumor when evaluating soft-tissue mass in the head and neck region.

## Highlights

• Pilomatrixoma, is a particular and uncommon type benign subcutaneous tumors arising from the cutaneous adnexa.
• Otolaryngologist should be familiar with this benign tumor when evaluating soft-tissue mass in the head and neck region.
• Clinical follow-up of these lesions is necessary to avoid recurrence and malignant transformation.

## 1 Introduction

Skin adnexal tumors are a heterogeneous group of neoplasms for which the diagnosis may be challenging, In an effort to simplify their classification, adnexal neoplasms into 3 groups: sebaceous, sweat gland-derived, and follicular [1].

Pilomatrixoma is a rare benign skin tumor differentiating toward hair matrix. It occurs as a firm nodule, most often on the face. Most occur in children and adolescents, but they can rarely occur in elderly patients [2]. We present a rare case of multiples pilomatrixomas in a 69-year-old patient. The clinical presentation, investigation and management are discussed.

This study has been reported in accordance with the SCARE criteria [3].

## 2 Case presentation

A sixty-nine year old male, farmer by profession, presented in our outpatient department with a 12-year history of multiple scalp masses. His past medical history was significant for diabetes mellitus. He stated that he did not consult beforehand, for fear of surgery.

**Fig. 1:**
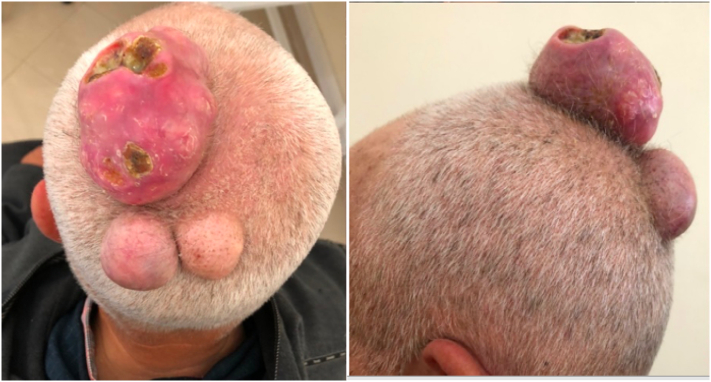
Preoperative appearance of the tumors.

Clinical examination revealed 3 subcutaneous bulky, rounded, firm masses of varying size on the parietal region. The largest one measured 17 × 9 cm and was ulcerated in places. Regional lymphadenopathy was absent (Fig. 1).

Head computed tomography (CT) showed 3 round, soft-tissue density lesions with calcifications.

**Fig. 2:**
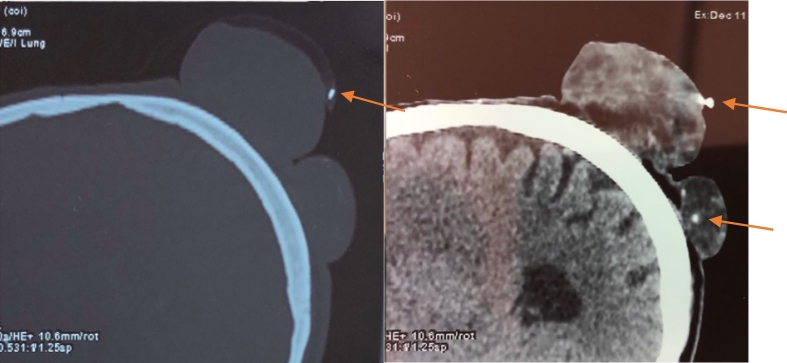
Coronal CT scan of the head showing the soft-tissue density lesions. Note the calcifications foci (arrows).

They was no evidence of osteolysis (Fig. 2).

The initial differential included malignant causes of scalp masses such as basal cell carcinoma, squamous cell carcinoma, metastasis and malignant adnexal tumors.

The patient underwent an incisional biopsy of the mass under local anesthesia of the largest mass.

Microscopically it consisted of multiple circumscribed islands of ghost cells and basaloid cells. Areas of ossification were noted. The histopathological pattern was consistent with a pilomatrixoma. The patient was scheduled for surgery.

**Fig. 3:**
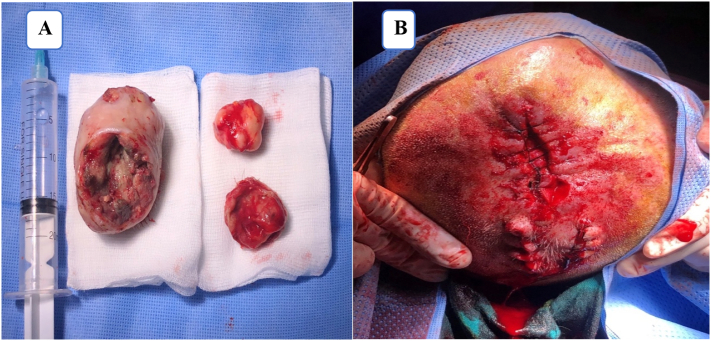
Composite operative picture. A: En bloc resection of the lesions. B: Immediate post-operative picture.

**Fig. 4:**
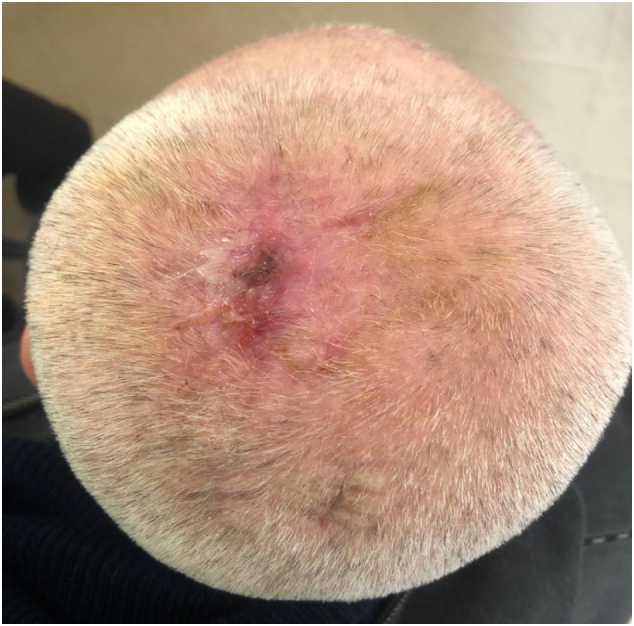
Post-operative picture at 4 months follow-up.

Under general anesthesia, the lesions were infiltrated with 2% lidocaine containing 1:10,000 norepinephrine. an elliptical incision was made over the parietal scalp masses. Skin was excised as the tumors were removed en bloc (Fig. 3). Using bipolar electrocautery adequate hemostasis was achieved. The whole surgical intervention was performed by a senior head and neck surgeon. The 2 small wounds were closed by direct suture without tension. The largest one was partially closed and daily dressing were performed until complete healing by secondary intention. (Fig. 4). Post-operative follow up was uneventful.

The patient was reevaluated at 3 months intervals by physical examination in our outpatient clinic. He showed no signs of recurrence.

## 3 Discussion

Pilomatrixoma or pilomatricoma, also known as calcifying epithelioma of Malherbe is a benign skin tumor of the hair follicule [4]. This pathology was first described by Malherbe and Chenantais in 1880 [5].

Pilomatrixoma is a rare disease, studies report the incidence to be between 0.001% and 0.0031% of all dermatohistopathologic materials submitted for examination [6]. Its peak incidence is in the first and second decades, but it can occur at any age. It Is slightly more common in women than men (ratio 1,15:1) [2].

The exact etiology and pathogenesis of pilomatrixoma are still unknown. Some studies have suggested that pilomatrixoma and pilomatrix carcinoma have mutations in the CTNNB1 gene, which encodes beta-catenin. This mutation leads to an upregulation of intracytoplasmic and intranuclear beta-catenin resulting in the development of neoplasms of hair matrix differentiation [7].

Several genetic syndromes have been associated with this disease, including MYH- associated polyposis, Sotos syndrome, myotonic dystrophy, gliomatosis cerebri; Rubinstein–Taybi syndrome, Gardner syndrome and Turner syndrome, which suggests alterations in other cell signaling pathways [2]. Concurrent disorders were not identified in our patient.

The lesions are most frequently single (94%) and of different size, ranging from 0,4 to 20 cm. They tend to be well-circumscribed, firm, and bluish with or without epidermal ulceration [2].

Stretching of the skin over the tumor shows the “tent sign” with multiple facets and angles, which appear to be a pathognomonic sign for pilomatricoma [8]. However, this sign is not commonly used as a diagnostic tool [2]. Head and neck are sites of predilection for this tumor, with up to 40% of cases occurring on the head alone. Other locations include upper limbs, trunk and lower limbs [9].

In the literature, ultrasound is the commonest imaging modality used for aid diagnosis. Pilomatrixomas appear as well-defined, ovoid, heterogeneous, hypoechoic masses, with internal echogenic foci; sign of calcification and posterior acoustic shadowing located in the skin [2].

CT scans can also be helpful in the diagnosis. Typical features of pilomatrixomas include a well-defined subcutaneous mass with mild to moderate enhancement and varying amounts of calcification [10]. This is consistent with our findings.

Histopathological examination of pilomatrixoma shows clearly delineated dermal nodules surrounded by a fibrous capsule located in the lower dermis and the subcutaneous fat. The cells are arranged in a circular pattern: basaloid cells on the periphery and enucleated shadow cells, also called ghost cells in the center. The basaloid cells exhibit both deeply staining nuclei with scant cytoplasm. The ghost cells, represent dead cells that show a central unstained area that corresponds to the lost nucleus. The transitional cells, are located between basaloid and ghost cells [11]. The incidence of calcification ranges from 69% to 85%. Reactive inflammatory foreign body giant cells, signifying a granulomatous response to the shadow cells and keratinized debris, may also be present [2]. Few cases of malignant transformation have been reported in the literature, typical features of pilomatrix carcinoma include proliferating basaloid cells with atypical mitoses and nuclear pleomorphism and invasion of underlying structures [11].

As performed in this case, the treatment of choice of this pathology is complete excision with clear margins [12]. The overlying skin must be included if the tumor is adherent to it. This is because malignant transformation is possible and spontaneous regression of pilomatrixoma has never been observed [13]. In cases of multiple pilomatrixomas, all lesions should be removed in the same procedure for the reasons mentioned above. The presence of familial or associated diseases must be taken into account. The incidence of recurrences after surgery has been reported to be between 0% and 6% [2].

## 4 Conclusion

Pilomatrixoma is a rare benign skin neoplasm differentiating toward hair matrix cells. It is more common in children and young adults. Complete surgical excision with clear margins remains the treatment of choice. Multiple pilomatrixomas should raise the suspicion of an underlying etiology.

## Declaration of compmeting interest

The authors of this article have no conflict or competing interests. All of the authors approved the final version of the manuscript.

## Funding

No funding was obtained for this study.

## Ethical approval

Written informed consent was obtained from the patient for publication of this case report and accompanying images. A copy of the written consent is available for review by the Editor-in-Chief of this journal on request.
